# Efficacy, safety and cost-effectiveness of obinutuzumab in patients with follicular lymphoma: a rapid review

**DOI:** 10.3389/fphar.2024.1426772

**Published:** 2025-01-03

**Authors:** Chao Wang, Yunzhuo Dong, Peng Men, Ruixia Zhang, Ying Xiao, Yishan Bu, Yinpeng Qin, Xinran Zhang, Qianqian Dou, Yiheng Yang, Huier Gao, Yi Zhang

**Affiliations:** ^1^ Department of Pharmacy, Tianjin First Central Hospital, Tianjin, China; ^2^ Department of Pharmacy, Peking University Third Hospital, Beijing, China

**Keywords:** obinutuzumab, follicular lymphoma, anti-CD20 monoclonal antibody, rapid review, efficacy, safety, cost-effectiveness

## Abstract

**Background:**

Obinutuzumab was approved in China in June 2021 used in combination with chemotherapy (followed by obinutuzumab maintenance) for the treatment of adult patients with previously untreated stage II bulky, III, or IV follicular lymphoma (FL). The clinical application of obinutuzumab has recently begun in China, but there is a lack of evidence to determine under which circumstances it should be considered the treatment of choice. A comprehensive assessment is necessary to evaluate the efficacy, safety, and cost-effectiveness of obinutuzumab in adult patients with FL.

**Objective:**

To summarize the evidence on the efficacy, safety, and cost-effectiveness of obinutuzumab in adult patients with FL, aiming to provide medical professionals with evidence for informed choices in clinical practice.

**Methods:**

The approach to this evidence synthesis was a rapid review of systematic reviews/meta-analyses (SR/meta-analyses), health technology assessment (HTA) reports, and pharmacoeconomic studies that brings together and summarizes the efficacy, safety, and cost-effectiveness of obinutuzumab in adult patients with FL. A literature search was conducted across multiple databases, including PubMed, Embase, Wanfang, CNKI, Weipu database, the Cochrane Library, the Centre for Reviews and Dissemination (CRD) database, International Network of Agencies for Health Technology Assessment (INAHTA) and Canada’s Drug Agency (CDA-AMC), International Society for Pharmacoeconomics and Outcomes Research (ISPOR), National Institute For Health and Care Excellence (NICE), Institute For Clinical And Economic Review (ICER), Grey Literature Database and Grey Net International. The studies on obinutuzumab for FL were searched in full text with *obinutuzumab, systematic review, meta-analysis, economics, cost, and health technology assessment* as keywords, with a search time frame from the date of database creation to 29 November 2024. The literature was screened based on predefined inclusion and exclusion criteria, and data were meticulously extracted and synthesized by two authors. Simultaneously, the quality of the literature was thoroughly assessed.

**Results:**

Obinutuzumab based chemotherapy (the chemotherapy regimen-cyclophosphamide, doxorubicin, vincristine, and prednisone (CHOP); cyclophosphamide, vincristine, and prednisone (CVP); or bendamustine) significantly prolonged progression free survival (PFS) compared to other chemotherapy regimen at primary and updated analyses. The incidence of grade 3–5 AEs, infusion-related reactions (IRRs), and infection were higher in the obinutuzumab based chemotherapy group compared to other chemotherapies. The economic researches conducted in China, United States, Japan, Italy and Norway had demonstrated that obinutuzumab-based chemothrepy was cost-effective compared to other chemothrepies. Although obinutuzumab significantly prolonged PFS and was cost-effective, its safety profile was considered lower.

**Conclusion:**

Compared with other chemothrapy regimen, obinutuzumab based chemotherapy significantly prolonged PFS and was cost-effective, while its safety profile was considered lower. Therefore, medical professionals should be caution when using or introducing obinutuzumab treatment for FL patients.

## 1 Introduction

FL is the most common indolent lymphoproliferative disorder (iNHL) and the second most frequent histological subtype among non-Hodgkin lymphomas after diffuse large B-cell lymphoma in western Europe (Esposito et al., 2023; Dreyling et al., 2021). FL arises within germinal centers and is characterized by the presence of the t (14; 18) translocation, which leads to aberrant BCL2 expression. Neoplastic cells express CD-20, CD-10, BCL2, and BCL6 through immunohistochemical staining (Merryman et al., 2024). Therefore, treatment of FL with monoclonal antibodies targeting CD-20 has proven to be an effective therapeutic option (Avilés et al., 2001). Rituximab was the first anti-CD20 monoclonal antibody to be licensed for use in hematological malignancies and has been in clinical use for over 20 years. Its integration into standard care has significantly improved outcomes in iNHL, particularly FL. Today, FL has a median survival exceeding 10 years, with older patients experiencing life expectancies comparable to age-matched healthy controls. However, resistance to rituximab is widely reported, which is defined as either a lack of response or clinical progression within 6 months after receiving a regimen containing rituximab. Furthermore, patients with iNHL, including FL, who relapse after single-agent therapy with rituximab show only a 40% response rate to retreatment with rituximab. This need to improve outcomes drives ongoing searches for novel therapies (O’Nions and Townsend, 2019).

Obinutuzumab, a new type II, glycoengineered, humanized anti-CD20 monoclonal antibody, has demonstrated longer PFS compared with rituximab (Davies et al., 2022; Marcus et al., 2017). Obinutuzumab was first marketed in the United States in 2016. The United States Food and Drug Administration approved obinutuzumab in combination with chemotherapy for previously untreated FL, based on the results of the GALLIUM trial (Marcus et al., 2017; Food and Drug Administration, 2016). In this trial, 1,202 patients were randomly assigned to receive either obinutuzumab based chemotherapy or rituximab based chemotherapy as induction treatment. Patients who responded positively underwent maintenance treatment for up to 2 years with the same antibody used during induction. The primary endpoint was investigator-assessed PFS. After a median follow-up of 34.5 months, the planned interim analysis demonstrated that obinutuzumab based chemotherapy significantly reduced the risk of progression, relapse, or mortality compared with rituximab based chemotherapy [estimated 3-year PFS rate: 80.0% vs. 73.3%; hazard ratio (HR) for progression, relapse, or death: 0.66; 95% confidence interval (CI): 0.51–0.85, *p* = 0.001]. Similar results were also seen regarding independently reviewed PFS and other time-to-event endpoints (Marcus et al., 2017). The 7-year outcomes further showed improved PFS in patients receiving obinutuzumab based chemotherapy compared with those receiving rituximab based chemotherapy (7-year PFS: HR = 0.77, 95% CI: 0.64–0.93, *p* = 0.0006), demonstrating the clinically meaningful and durable benefit of obinutuzumab based chemotherapy in previously patients with untreated FL (Townsend et al., 2023).

Obinutuzumab is also approved for treating patients with FL who are refractory to or have relapsed following rituximab therapy (Food and Drug Administration, 2016). In an open-label, randomized phase 3 study (GADOLIN), 413 patients with rituximab-refractory iNHL, including 335 with FL, were randomly assigned to receive obinutuzumab in combination with bendamustine, followed by obinutuzumab maintenance or bendamustine monotherapy. The interim analysis showed that the obinutuzumab arm had significantly longer median PFS, confirmed in an updated analysis that demonstrated a PFS benefit (HR = 0.57, 95% CI: 0.44–0.73, *p* < 0.001), as well as longer median overall survival (OS) (HR = 0.67, 95% CI: 0.47–0.96, *p* = 0.027) (Sehn et al., 2016; Cheson et al., 2018). PFS and OS benefits were similar in patients with FL (Cheson et al., 2018). Furthermore, current guidelines recommend obinutuzumab as first-line treatment for patients when the treatment goals are complete remission and prolonged PFS (Ma, 2021; Zhu et al., 2021; McNamara et al., 2020; National Institute for Health and Care Excellence, 2020; National Institute for Health and Care Excellence, 2018). However, obinutuzumab has a higher incidence of grade 3–5 AEs, particularly IRRs and neutropenia, compared with other treatments. This has been observed in both randomized controlled trials (RCTs) (Marcus et al., 2017; Sehn et al., 2016) and real-world studies (Berger et al., 2023; Claustre et al., 2023). Regarding the cost of treatment, a real-world study provided an update on healthcare utilization and costs among patients initiating first-line treatment for FL as recommended by the National Comprehensive Cancer Network (NCCN) in the United States. Unadjusted 6-month total healthcare costs were highest with rituximab plus bendamustine ($174,407), followed by obinutuzumab plus bendamustine ($163,548), while the lowest costs were observed with rituximab-CVP ($91,762) and rituximab monotherapy ($89, 201) (Ta et al., 2021).

Obinutuzumab was also approved in China in June 2021 for use in combination with chemotherapy (followed by obinutuzumab maintenance) to treat adult patients with previously untreated stage II bulky, III, or IV FL (Food and Drug Administration, 2016). However, the Chinese guidelines for the diagnosis and treatment of follicular lymphoma (Ma, 2021; Chinese Society of Lymphoma, 2020) point out that obinutuzumab can also be used in patients with R/R-rituximab FL. Nevertheless, there is insufficient high-quality evidence to make a recommendation.

The clinical application of obinutuzumab in FL patients has recently begun in China.

Preliminary scoping of the literature revealed a lack of evidence to determine under which circumstances it should be considered the best treatment of choice compared with the classical chemotherapy regimens. To address this, we conducted a rapid review to identify the evidence about the efficacy, safety, and cost-effectiveness of obinutuzumab in adult patients with FL, aiming to provide medical professionals with evidence to support informed clinical choices.

## 2 Methods

### 2.1 Types of study design

The approach to this evidence synthesis was a rapid review (Guo et al., 2024; Garritty et al., 2024; Burrows et al., 2022; Spiers et al., 2021; Haby et al., 2016) of systematic reviews/meta-analyses, HTA reports and pharmacoeconomic studies. Preliminary scoping of the literature revealed a lack of evidence for obinutuzumab to determine under which circumstances it should be considered the best treatment of choice compared with the classical chemotherapy regimens. Rapid review methodology was employed, which uses a streamlined approach to study selection and synthesis in order to produce a timely overview of evidence for medical professionals. The following methods are reported in accordance with the Preferred Reporting Items for Systematic Reviews and Meta-Analyses guidelines (https://www.prisma-statement.org/).

### 2.2 Search strategy

We conducted a comprehensive search of multiple databases, including PubMed, Embase, Wanfang, CNKI, Weipu database, the Cochrane Library, the CRD database (https://www.crd.york.ac.uk/CRDWeb/), INAHTA-International HTA Database (https://database.inahta.org/), CDA-AMC (https://www.cda-amc.ca), ISPOR (https://www.ispor.org/), NICE (https://www.nice.org.uk/), ICER (https://icer.org/), Grey Literature Database (https://opengrey.eu/) and Grey Net International (https://www.greynet.org/home.html) to identify relevant studies. The relevant studies on obinutuzumab for FL were searched in full text with *obinutuzumab, systematic review, meta-analysis, economics, cost*, and *health technology assessment* as keywords, with a search time frame from the date of database creation to 29 November 2024. References to included literature relevant to our study have been retrived and supplemented to ensure the comprehensiveness of the search.

### 2.3 Inclusion of exclusion criteria

#### 2.3.1 Inclusion criteria

Inclusion criteria are as follows: 1) types of studies: SR/meta-analyses, HTA reports and pharmacoeconomic studies with no language restrictions; 2) participants: the included studies focused on adult human populations (aged 18 years or older) who had been diagnosed with FL; 3) interventions/comparison: the included studies with the intervention consisted of obinutuzumab based chemotherapy, while the control group received either rituximab based chemotherapy or other chemotherapy regimens; 4) outcomes: the included studies with the primary efficacy endpoints including PFS, OS, and objective response rate (ORR), or those with the safety outcomes such as the incidence of all grade AEs, grade 3–5 AEs, IRRs, neutropenia, thrombocytopenia and infection, as well as some publications that inlcuded health economic components with pharmacoeconomic outcome like ICER.

#### 2.3.2 Exclusion criteria

The exclusion criteria were as follows: 1) studies involving non-obinutuzumab treatments in FL patients, studies on the use of obinutuzumab in non-FL indications; 2) unavailable full text; 3) conference abstracts, literature reviews, letters, editorials, duplicate publications; 4) studies about animal experiments of obinutuzumab; and 5) primary clinical studies of obinutuzumab, including RCTs, controlled clinical trials, observational studies (cohort studies, case reports or case series) and *in vitro* studies. As for health economic publications, researches that does not focus on the cost-effectiveness analysis, such as cost-minimization analysis, cost-benefit analysis were also excluded.

### 2.4 Literature screening

After conducting a literature deduplication process, two researchers (Chao Wang and Yunzhuo Dong) meticulously screened and cross-verified the titles, abstracts, and full texts based on predefined inclusion and exclusion criteria. In case of any discrepancies, they engaged in discussions with a third researcher (Peng Men).

### 2.5 Data extraction

Data were extracted according to the pre-designed data extraction table, including author, publication year, study type, research methods, population, sample size, intervention/control measures and outcomes by two researchers.

### 2.6 Quality evaluation

The HTA checklist developed by the INAHTA was utilized to evaluate the quality of HTA reports (International Network of Agencies for Health Technology Assessment, 2014). A Measurement Tool to Assess Systematic Reviews (AMSTAR-2) was applied to evaluate methodological quality of eligible SR/meta-analyses (Shea et al., 2017). The quality of pharmacoeconomic researches were evaluated using Consolidated Health Economic Evaluation Reporting Standards (CHEERS) (Husereau et al., 2022). The quality of all included literature was assessed and double-checked by two investigators (Chao Wang and Yunzhuo Dong) utilizing these diverse tools. We resolved the disagreements by consulting a third reviewer (Peng Men).

### 2.7 Data analysis

The HTA reports, SR/meta-analyses, and pharmacoeconomic studies included in this study were narratively analyzed based on the characteristics of different populations, interventions, and outcomes. Summary tables were produced to present key data from the included studies.

## 3 Results

### 3.1 Results of literature search/screening

**FIGURE 1:**
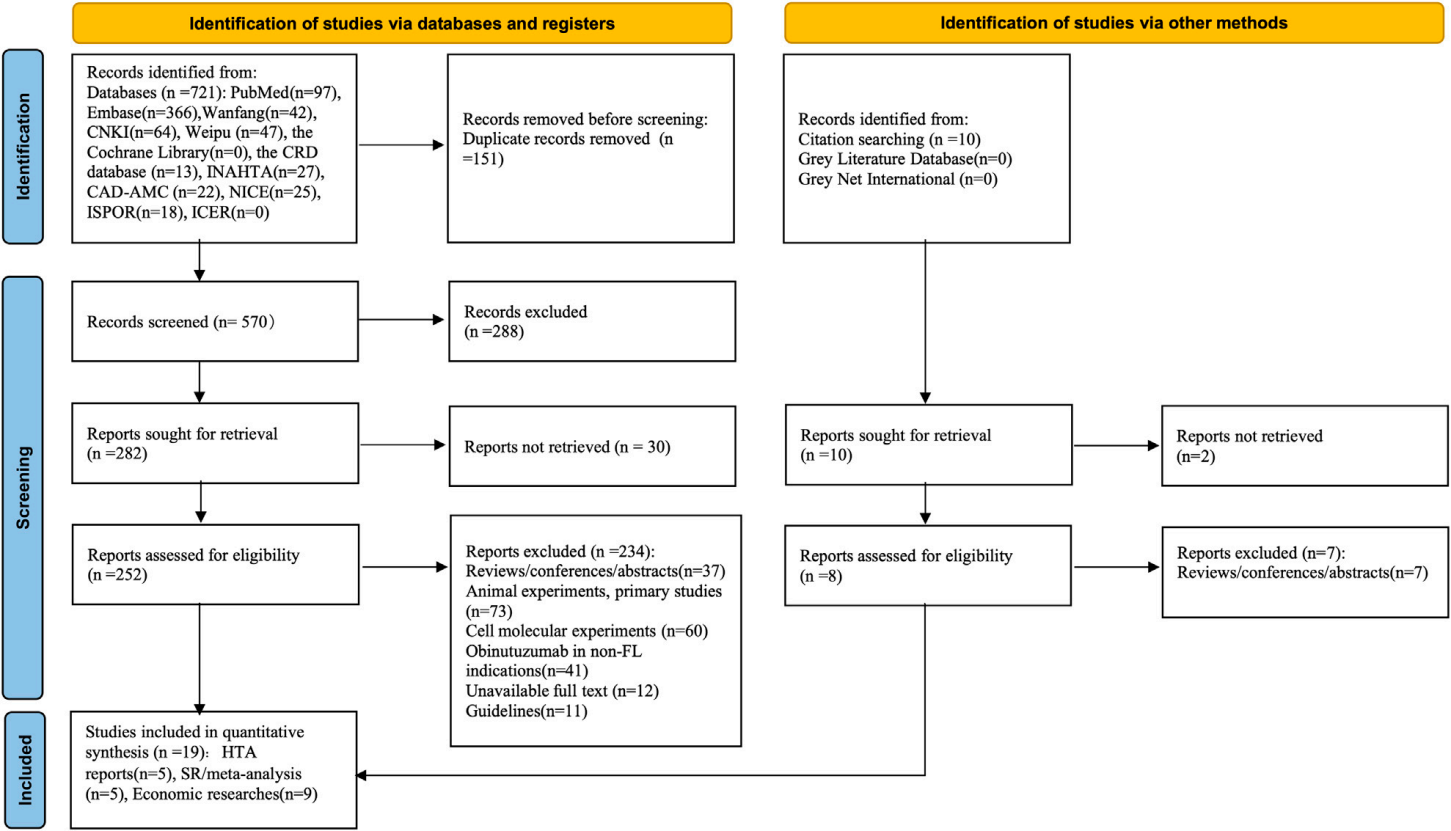
Flow diagram for included researches of databases and registers.

A total of 731 studies were retrieved from various databases according to the search strategy devised by two researchers. After eliminating duplicates and conducting preliminary screening based on titles and abstracts, 260 sources proceeded to the full-text review stage. Ultimately, 19 studies were included in this study following a thorough examination of the full texts. These comprised five published HTA reports, five SR/meta-analyses, and nine pharmacoeconomic studies. The retrieval and screening process is detailed in Figure 1.

### 3.2 Characteristics and quality evaluation of the included literature

The characteristics of the included studies are shown in Tables 1–3. The five HTA reports (Pan-Candian Oncology Drug Review, 2018; Scottish Medicines Consortium, 2018; Pan-Candian Oncology Drug Review, 2017; Horizon Scanning in Oncology, 2016; PBAC meeting, 2016) which were mainly retrived from HTA database (CRD database, INAHTA-International HTA Database, CAD-AMC) originated from Canada, the United Kingdom, Austria, and Australia. These studies comprehensively evaluated the efficacy, safety and economic characteristics of obinutuzumab. The primary data for these reports were derived from two open-label, multicenter, randomized phase III clinical trials, GALLIUM and GADOLIN. The characteristics of the five included SRs/meta-analyses are presented in Table 2 (Leng, 2023; Chu et al., 2024; Wang et al., 2022; Amitai et al., 2021; Police et al., 2016). We extracted the outcomes of one study through indirect comparisons involving obinutuzumab (Police et al., 2016), while the others were all direct comparisons (Leng, 2023; Chu et al., 2024; Wang et al., 2022; Amitai et al., 2021). All pharmacoeconomic studies were cost-effectiveness analyses conducted in China, Italy, the US, Japan, Norway, and Ireland. All of these studies adapted partitioned survival models. The characteristics of these studies are shown in Table 3 (Ma et al., 2023; Wei and Liu, 2022; Bellone et al., 2021; Spencer et al., 2021; Ohno et al., 2020; Guzauskas et al., 2019; Haukaas et al., 2018; Guzauskas et al., 2018; National Centre for Pharmacoeconomics, 2018).

**TABLE 1:** The characteristics and outcomes of HTA reports.

Country/institution	Publication time	Population	Intervention/control measures	Outcomes
Canada/pCODR	2018	Previously untreated FL patients	Obinutuzumab based chemotherapy VS. rituximab based chemotherapy	3-year investigator-assessed PFS: HR = 0.66, (95% CI: 0.51–0.85), *p* = 0.0012, primary analysis; HR = 0.68, (95%CI: 0.54–0.87), *p* = 0.0016) updated analysis 3-year independent review committee assessed PFS: HR = 0.71, (95% CI: 0.54–0.93), *p* = 0.0138, primary analysis; HR = 0.72, (95% CI: 0.56–0.93), *p* = 0.012, updated analysis 3-year OS: HR = 0.75, (95% CI: 0.49–1.17), *p* = 0.21, primary analysis; HR = 0.82, (95% CI: 0.54–1.22), *p* = 0.32),updated analysis ORR: RD = 1.6, (95% CI: -2.1-5.5) All-grade and grade 3–5 AEs: 99.5% vs. 98.3%, 74.6% vs. 67.8% All-grade IRRs and Grade 3–5 IRRs: 68.2% VS. 58.5%; 12.4% VS. 6.7% All-grade Neutropenia and Grade 3–5 Neutropenia: 50.6% VS. 45.1%; 45.9% VS. 39.5 All-grade thrombocytopenia and grade 3–5 thrombocytopenia: 11.4% vs. 7.5%; 6.1% vs. 2.7% All-grade infection and Grade 3–5 infection: 77.3% VS. 70%; 20.0% VS. 15.6%
Scotland/SMC	2018	Previously untreated advanced FL patients	Obinutuzumab based chemotherapy VS. rituximab based chemotherapy	3-year investigator-assessed PFS:HR = 0.66, (95% CI: 0.51–0.85), *p* = 0.0012 3-year independent review committee assessed PFS: HR = 0.71, (95% CI: 0.54–0.93), *p* = 0.0138 3-year OS: HR = 0.75, (95% CI: 0.49–1.17), *p* = 0.21 Grade 3–5 AEs: 75% vs. 68% Grade 3–5 IRR: 12% VS. 6.7% Grade 3–5 Neutropenia: 46% VS. 40% Grade 3–5 infection: 20.0% VS. 15.6% Grade 3–5 thrombocytopenia: 6.1% vs. 2.7%
Canada/pCODR	2017	R/R-rituximab FL patients	Obinutuzumab plus bendamustine VS. bendamustine	Independent review committee assessed PFS: HR = 0.48, (95% CI: 0.34–0.68), *p* < 0.0001, first efficacy analysis; HR = 0.47, (95% CI: 0.34–0.64), *p* < 0.0001, second efficacy analysis; HR = 0.52 (95%CI: 0.39–0.69), *p* < 0.0001, third efficacy analysis OS: HR = 0.71, (95% CI: 0.43–1.19), *p =* 0.02, first efficacy analysis; HR = 0.62, (95% CI: 0.39–0.98),*p* = 0.038, second efficacy analysis; HR = 0.58, (95% CI: 0.39–0.86), *p* = 0.0061, third efficacy analysis All-grades and grade 3–5 AEs: 43.6% VS. 36.9%; 72.5% VS. 65.5%, third safety analysis Grade 3–5 IRRs: 9.3% VS. 3.4%, third safety analysis Grade 3–5 neutropenia: 34.5% VS. 27.1%, third safety analysis Grade 3–5 thrombocytopenia: 10.8% VS. 15.8%, third safety analysis Grade 3–5 infection: 22.5% VS. 19.2%, third safety analysis
Austria/Ludwig Boltzmann Institute	2016	R/R-rituxiamb FL patients	Obinutuzumab plus bendamustine VS. bendamustine	Independent review committee assessed PFS: HR = 0.48, (95% CI: 0.34–0.68), *p* < 0.0001 Investigator-assessed PFS: HR = 0.48, (95% CI: 0.35–0.67), *p* < 0.0001 All grades and grade 3–5 IRRs: 69% VS. 63%, 11% VS. 6%, first safety analysis All grades and grade 3–5 neutropenia: 35% VS. 25%, 33%VS.26%, first safety analysis Grade 3–5 thrombocytopenia: 10.8% VS. 16.2%, first safety analysis
Australia /PBAC	2016	R/R-rituxiamb FL patients	Obinutuzumab plus bendamustine VS. bendamustine	PFS: HR = 0.48, (95%CI:0.34–0.68), *p* < 0.0001, first efficacy analysis; HR = 0.47, (95% CI: 0.34–0.64), *p* < 0.0001, second efficacy analysis OS: HR = 0.71, (95% CI: 0.43–1.19), *p* = 0.02, first efficacy analysis; HR = 0.62, (95% CI: 0.39–0.98), *p* = 0.038, second efficacy analysis Grade 3–5 IRRs: RD = 5.6%, (95%CI: 0.3%–10.8%), second safety analysis Grade 3–5 neutropenia: RD = 7.9%, (95%CI: -1.8%-17.6%), second safety analysis

PFS, prolonged progression free survival; OS, overall survival; ORR, objective response rate.

**TABLE 2:** The characteristics and outcomes of included systematic review/meta-analysis.

Study ID	Population	Intervention/control measures	The number of studies associated with obinutuzumab	The total cases associated with obinutuzumab	Outcomes
Leng WT 2023	FL patients	Obinutuzumab based chemotherapy VS. rituximab based chemotherapy	19	1,684	PFS: HR = 0.75, (95% CI: 0.58–0.96), *p* = 0.03 2-year OS rate: 90% (95% CI: 84–95) ORR: OR = 1.30, 95% CI: 0.96–1.75, *p* = 0.09) ≧Grade 3 IRRs rate: 7% (95% CI: 2–15) ≧Grade 3 neutropenia rate: 35% (95% CI: 26–44) ≧Grade 3 thrombocytopenia rate:8% (95% CI: 4–12) ≧Grade 3 infection rate: 20% (95% CI: 18–23)
Chu YR 2023	Previously untreated advanced FL patients	Obinutuzumab based chemotherapy VS. rituximab based chemotherapy	1	601	PFS: HR = 0.43, (95% CI: 0.22–0.79) OS: HR = 0.43, (95% CI: 0.18–1.90)
Wang YC 2022	FL patients	Obinutuzumab based chemotherapy VS. rituximab based chemotherapy	1	601	PFS: HR = 0.68 (95% CI: 0.54–0.87), *p* = 0.0016
		Obinutuzumab plus bendamustine VS. rituximab plus bendamustine	1	345	PFS: HR = 0.63 (95% CI: 0.46–0.88), *p* = 0.0062
		Obinutuzumab plus CHOP VS. rituximab plus CHOP	1	196	PFS: HR = 0.72 (95% CI: 0.48–1.10), *p* = 0.13
		Obinutuzumab plus CVP VS. rituximab plus CVP	1	60	PFS: HR = 0.79 (95% CI: 0.42–1.47), *p* = 0.46
Amitai I 2021	FL, chronic lymphocytic leukemia, and diffuse large B-cell lymphoma	Obinutuzumab based chemotherapy VS. rituximab based chemotherapy	2	675	All grade AEs: RR = 1.05, (95% CI: 1.00–1.10), *p >* 0.05 Grade 3–4 AEs: RR = 1.15, (95% CI: 1.09–1.20), *p* < 0.05 Grade 3–4 IRRs: RR = 2.8, (95% CI: 2.16–3.64), *p* = 0.02 Grade 3–4 neutropenia: RR = 1.17, (95% CI: 1.0–1.36), *p* < 0.00001 Grade 3–4 thrombocytopenia: RR = 2.8, (95% CI: 1.92–4.06), *p* < 0.00001
Police RL 2016	R/R-rituximab FL patients	rituximab plus bortezomib VS. obinutuzumab	1	88	ORR: RR = 1.14, 95% CI: 0.76–1.72, *p* > 0.05
		rituximab + sargasstim VS. obinutuzumab	1	88	ORR: RR = 0.92, 95% CI: 0.50–1.65, *p* > 0.05

PFS, prolonged progression free survival; OS, overall survival; ORR, objective response rate.

**TABLE 3:** The characteristics of included pharmacoeconomic studies.

Study ID	Country	Economic research methods	Perspective	Population	Time horizon	Intervention versus control measures
Ma J 2023	China	Cost-utility analysis	Healthcare payers	R/R-rituximab FL patients	life time	Obinutuzumab plus bendamustine VS. bendamustine
Wei SD 2022	China	Cost-utility analysis	Healthcare perspective	Previously untreated advanced FL patients	32 years	Obinutuzumab plus CHOP VS. rituximab plus CHOP
BelloneM 2021	Italy	Cost-utility analysis	The Italian National Health Service	Previously untreated advanced FL patients	Lifetime	Obinutuzumab based chemotherapy VS. rituximab based chemotherapy
Spencer SJ 2021	United States	Cost-utility analysis	U.S. healthcare perspective	Previously untreated advanced FL patients	Lifetime	Obinutuzumab based chemotherapy VS. rituximab (Ra/Rb) based chemotherapy
Ohno S 2020	Japan	Cost-utility analysis	The payer’s perspective	Previously untreated advanced FL patients	Lifetime	Obinutuzumab based chemotherapy VS. rituximab based chemotherapy
Guzauskas GF 2019	United States	Cost-utility analysis	U.S. payer perspective	Previously untreated advanced FL patients	Lifetime	Obinutuzumab based chemotherapy VS. rituximab based chemotherapy
Haukaas SF 2018	Norway	Cost-utility analysis	Norwegian health payer perspective	R/R-rituximab FL patients	20 years	Obinutuzumab plus bendamustine VS. bendamustine
Guzauskas GF 2018	United States	Cost-utility analysis	U.S. payer perspective	R/R-rituximab FL patients	Lifetime	Obinutuzumab plus bendamustine VS. bendamustine
NCPE 2018	Ireland	Cost-utility analysis	The payer’s perspective	Previously untreated advanced FL patients	50 years	Obinutuzumab based chemotherapy VS. rituximab based chemotherapy

The methodological quality evaluation of the included HTA reports is summarized in Supplementary Table A1. The overall quality of reporting of the included studies ranges from good to poor. Two HTAs [SMC 2018 (Scottish Medicines Consortium, 2018) and PBAC 2016 (PBAC meeting, 2016)] had some key information not reported, which prevented us from giving high-quality scores based on the publicly available reports. The primary issue with Scottish Medicines Alliance (SMC) 2018 (Scottish Medicines Consortium, 2018) was the absence of a clear process for evidence production and inadequate explanation of the context for data assessment and interpretation. The main factors contributing to the downgrade in the study submitted by the manufacturer to Pharmaceutical Benefits Advisory Committee (PBAC) 2016 (PBAC meeting, 2016) were deficiencies in items 1–4 under “Preliminary Information” and issues in the “Assessment Process” domain. The AMSTAR-2 evaluation revealed that most of the included SRs/meta-analyses were of good quality (Supplementary Table A2). All pharmacoeconomic studies the overall quality was generally good, except for the National Centre for Pharmacoeconomics (NCPE) summary (National Centre for Pharmacoeconomics, 2018) from Ireland, which had numerous items on the CHEERS checklist that could not be evaluated. The overall compliance rate for the CHEERS checklist in NCPE 2018 (National Centre for Pharmacoeconomics, 2018) was 58.33%. The main areas of concern were a lack of detailed information in the “*Methodology*” and “*Results*” sections, as well as missing details regarding “*Source of Funding*” and “*Conflicts of Interest*” (Supplementary Table A3).

### 3.3 Effectiveness evaluation

#### 3.3.1 Effect of obinutuzumab on PFS in patients with FL

Two HTA reports analyzed the effect of obinutuzumab based chemotherapy compared with rituximab based chemotherapy and found a significant prolongation of 3-year PFS in previously untreated FL patients (*p* < 0.01) (Pan-Candian Oncology Drug Review, 2018; Scottish Medicines Consortium, 2018). The efficacy of obinutuzumab plus bendamustine in prolonging PFS was superior to rituximab plus bendamustine (HR = 0.63, 95% CI: 0.46–0.88, *p* = 0.0062). However, there was no significant difference between obinutuzumab plus chemotherapy (CHOP) and rituximab plus chemotherapy (CHOP) in terms of PFS prolongation, or between obinutuzumab plus chemotherapy (CVP) and rituximab plus chemotherapy (CVP) (*p* > 0.01) (Wang et al., 2022). Additionally, three HTA reports (Pan-Candian Oncology Drug Review, 2017; Horizon Scanning in Oncology, 2016; PBAC meeting, 2016) demonstrated that obinutuzumab plus bendamustine compared with bendamustine monotherapy significantly extended the independent review committee assessed PFS and investigator-assessed PFS at primary and updated analyses in R/R-rituximab FL patients (*p* < 0.0001). Three recent meta-analyses indicated that, based on PFS, obinutuzumab outperformed other treatments, these findings were consistent with the HTA reports (Leng, 2023; Chu et al., 2024; Wang et al., 2022). The 2-year PFS rate in the obinutuzumab based chemotherapy regimen was found to be 74% (95% CI: 65–82) (Leng, 2023) (Tables 1, 2).

Based on the current studies, obinutuzumab based chemotherapy could effectively prolong the PFS of both previously untreated and R/R-rituximab FL patients compared to other chemothrapies.

#### 3.3.2 Effect of obinutuzumab on OS in patients with FL

The comparison between obinutuzumab based chemotherapy and rituximab based chemotherapy did not show a significant improvement in 3-year OS (*p >* 0.05) (Pan-Candian Oncology Drug Review, 2018; Scottish Medicines Consortium, 2018). Such results were in accordance with a recently published network meta-analysis (Chu et al., 2024). However, obinutuzumab plus bendamustine showed a significant improvement in OS compared with bendamustine in the updated analyses (HR = 0.62, 95% CI: 0.39–0.98, *p =* 0.038; HR = 0.58, 95% CI: 0.39–0.86, *p* = 0.0061) (Pan-Candian Oncology Drug Review, 2017; PBAC meeting, 2016). The 2-year OS rate was 90% (95% CI: 84–95) (Leng, 2023) (Tables 1, 2).

Based on the findings, obinutuzumab plus bendamustine could provided a prolongation of OS compared with bendamustione in R/R-rituximab FL patients.

#### 3.3.3 Effect of obinutuzumab on ORR in patients with FL

One HTA report analyzed the effect of obinutuzumab based chemotherapy compared with rituximab based chemotherapy on ORR at the end of the induction and found no difference between the two groups [rate difference (RD) = 1.6, 95% CI: -2.1-5.5, *p* = 0.33) (Pan-Candian Oncology Drug Review, 2018). Two additional meta-analyses similarly found no advantage of obinutuzumab in improving ORR compared with other chemotherapy regimens (*p* > 0.05) (Leng, 2023; Police et al., 2016). The 2-year ORR rate was 79% (95% CI: 73–85) (Leng, 2023) (Tables 1, 2).

Based on these evidence, obinutuzumab-based chemothrapy did not show any advantage in improving ORR compared with other chemotherapies for FL patients.

### 3.4 Safety evaluation

#### 3.4.1 All-grades and grade 3–5 AEs

Obinutuzumab based chemotherapy had a higher incidence of all-grades and grade 3–5 AEs than other chemotherapies (Pan-Candian Oncology Drug Review, 2018; Scottish Medicines Consortium, 2018). A meta-analysis also revealed that obinutuzumab based chemotherapy significantly increased the incidence of grade 3–4 AEs compared with rituximab based chemotherapy (RR = 1.15, 95% CI: 1.09–1.20, *p <* 0.05) (Amitai et al., 2021).

Despite the higher incidence of ≧grade 3 AEs associated with obinutuzumab, according to a recent network meta-analysis, obinutuzumab may be the best treatment option in terms of the benefit-risk ratio for grade ≥3 AEs (Chu et al., 2024) (Tables 1, 2).

#### 3.4.2 IRRs

Two studies demonstrated that obinutuzumab based chemotherapy resulted in a higher incidence of all grades and grade 3–5 IRRs than rituximab based chemotherapy (Pan-Candian Oncology Drug Review, 2018; Scottish Medicines Consortium, 2018). A meta-analysis also revealed that the occurrence of grade 3–4 IRRs was significantly higher in obinutuzumab based chemotherapy than in rituximab based chemotherapy (RR = 2.8, 95% CI: 2.16–3.64) (Amitai et al., 2021). Three HTA reports (Pan-Candian Oncology Drug Review, 2017; Horizon Scanning in Oncology, 2016; PBAC meeting, 2016) found a higher incidence of all grades and grade 3–5 IRRs in the obinutuzumab plus bendamustine group than in the bendamustine group in both the primary and updated analyses. Additionally, another meta-analysis reported an IRRs rate of 7% (95% CI: 2–15) (Leng, 2023) (Tables 1, 2).

Based on these evidence, obinutuzumab based chemotherapy may results in a high incidence of all-grade and grade 3–5 IRRs compared to other chemothrapy regimen in both previously untreated and R/R-rituximab FL patients.

#### 3.4.3 Neutropenia

Two studies demonstrated that obinutuzumab based chemotherapy had a higher incidence of all grade and grades 3–5 neutropenia than rituximab based chemotherapy (Pan-Candian Oncology Drug Review, 2018; Scottish Medicines Consortium, 2018). A meta-analysis revealed no statistically significant difference in the incidence of grade 3–4 neutropenia between obinutuzumab based chemotherapy and rituximab based chemotherapy regimens (RR = 1.01, 95% CI: 0.93–1.11) (Amitai et al., 2021). Three HTA studies found that the incidence of grade 3–5 neutropenia was high in obinutuzumab plus bendamustine compared to bendamustine, although this difference was not statistically significant in both primary and updated analyses (RD = 7.9%, 95% CI: -1.8-17.6) (Pan-Candian Oncology Drug Review, 2017; Horizon Scanning in Oncology, 2016; PBAC meeting, 2016). In another study, it was found that neutropenia was the most common hematological AE, with a prevalence of 35% (95% CI: 26–44) (Leng, 2023) (Tables 1, 2).

Thus, obinutuzumab based chemotherapy may results in a high incidence of all-grade and grade 3–5 neutropenia compared to other chemothrapies in both previously untreated and R/R-rituximab FL patients.

#### 3.4.4 Thrombocytopenia

One study described an increased incidence of all grades and grade 3–5 thrombocytopenia in patients receiving obinutuzumab based chemotherapy compared to rituximab based chemotherapy (Pan-Candian Oncology Drug Review, 2018). Another meta-analysis also showed that obinutuzumab based chemotherapy had a significantly higher incidence of grade 3–4 thrombocytopenia than rituximab based chemotherapy (RR = 2.8, 95% CI: 1.92–4.06, *p* < 0.00001) (Amitai et al., 2021). While the incidence of grade 3–5 thrombocytopenia was lower in the obinutuzumab plus bendamustine group than in the bendamustine group in primary and updated analyses, according to another two studies (10.8% vs. 16.2%; 10.8% vs. 15.8%) (Pan-Candian Oncology Drug Review, 2017; Horizon Scanning in Oncology, 2016). The thrombocytopenia rate was found to be 8% (95% CI: 4–12) in a recently published study (Leng, 2023) (Tables 1, 2).

Therefore, obinutuzumab based chemotherapy may result in a high incidence of all-grades and grade 3–5 thrombocytopenia compared with rituximab based chemotherapy in previously untreated FL patients.

#### 3.4.5 Infection

The incidence of grade 3–5 infection was found to be higher in patients receiving obinutuzumab based chemotherapy compared to those receiving other chemotherapies (Pan-Candian Oncology Drug Review, 2018; Scottish Medicines Consortium, 2018; Pan-Candian Oncology Drug Review, 2017). A meta-analysis also indicated that the obinutuzumab based chemotherapy exhibited a significantly higher incidence of grades 3–4 infection than rituximab based chemotherapy group (RR = 1.17, 95% CI: 1.0–1.36,*p* < 0.00001) (Amitai et al., 2021). Another study found that the most common non-hematological AE was infection, with a prevalence of 20% (95% CI: 18–23) (Leng, 2023) (Tables 1, 2).

Therefore, obinutuzumab based chemotherapy may results in a high incidence of grade 3–5 infection compared to other chemothrapies in both previously untreated and R/R-rituximab FL patients.

### 3.5 Economic evaluation

A total of six pharmacoeconomic studies compared the cost-effectiveness of obinutuzumab based chemotherapy and rituximab based chemotherapy in patients with previously untreated FL (Wei and Liu, 2022; Bellone et al., 2021; Spencer et al., 2021; Ohno et al., 2020; Guzauskas et al., 2019; National Centre for Pharmacoeconomics, 2018). The recent cost-effectiveness analysis in China showed that obinutuzumab plus CHOP for FL is considered cost-effective compared with rituximab plus CHOP from a healthcare perspective, regardless of whether the original rituximab drug or rituximab biosimilar drug was chosen, when obinutuzumab was priced at approximately CNY13,760 (the new Medicare threshold) (Wei and Liu, 2022). From the perspective of the Italian NHS, a cost-utility analysis in Italy showed that the ICER of €17,000/QALY was lower than the threshold of Willingness to Pay in industrialized countries, obinutuzumab based chemotherapy was cost-effective option in first-line treatment of patients with advanced FL at intermediate or high risk (Bellone et al., 2021). The cost-utility analysis conducted in Japan, from the payer’s perspective, showed that the ICER were lower than the threshold for cancer treatments in the country, implying that obinutuzumab based chemotherapy was cost-effective treatment regimen for Japanese patients with previously untreated FL (Ohno et al., 2020). From the perspective of US payers, Guzauskas et al. (2019) demonstrated that treatment with obinutuzumab based chemotherapy was cost-effective for patients with previously untreated FL in the US compared with rituximab based chemotherapy, with an ICER of $2300/QALY gained. In the cost-effectiveness analyses of two HTA reports (Pan-Candian Oncology Drug Review, 2018; Scottish Medicines Consortium, 2018), which were submitted by the manufacturer to the pan-Canadian Oncology Drug Review (pCODR) Economic Guidance Panel (EGP) (Pan-Candian Oncology Drug Review, 2018) and the SMC (Scottish Medicines Consortium, 2018), obinutuzumab based chemotherapy compared with rituximab based chemotherapy resulted in the base case ICERs of $49,562/QALY and €57,858/QALY, respectively. In the cost-effectiveness analysis of obinutuzumab for the first line treatment of FL submitted by the manufacturer to the NCPE, the ICER of obinutuzumab compared with rituximab was €53246/QALY.

The pCODR EGP (Pan-Candian Oncology Drug Review, 2018), the SMC (Scottish Medicines Consortium, 2018), and the NCPE (National Centre for Pharmacoeconomics, 2018) posed many restrictions and conservative assumptions, estimating that obinutuzumab based chemotherapy compared with rituximab based chemotherapy was limited cost-effectiveness.

We found that the journal publications considered obinutuzumab based chemothrapy to be cost-effective compared with rituximab based chemothrapy in previously untreated FL patients. However, in the economic evaluation submitted by the manufacturer, national pharmacoeconomic/or drug agencies from different countries drove for a conservative assessment.

A total of three studies (Ma et al., 2023; Haukaas et al., 2018; Guzauskas et al., 2018) compared the cost-effectiveness of obinutuzumab plus bendamustine with bendamustine in R/R-rituximab FL patients. In a recently published study in China, researchers used a decision-making model to find that obinutuzumab plus bendamustine was a cost-effective choice compared to traditional treatments for Chinese patients with R/R-rituximab FL (Ma et al., 2023). Another study compared the cost-effectiveness of obinutuzumab plus bendamustine with bendamustine from the Norwegian healthcare payer perspective and found that the ICER was €46,438/QALY below the acceptable threshold (€89,000/QALY). The results indicated that obinutuzumab plus bendamustine may be cost-effective compared with bendamustine in Norway (Haukaas et al., 2018). The cost-utility analysis conducted by Guzauskas et al. (2018), from the US payers perspective, showed that obinutuzumab plus bendamustine resulted in an ICER of about $47,000/QALY and was considered cost-effective at the $100,000/QALY threshold. However, the HTA reports on ecomonic evaluation, the pCODR EGP indicated that obinutuzumab plus bendamustine was not cost-effective compared to bendamustine monotherapy in Canada (Pan-Candian Oncology Drug Review, 2017). Another analysis submitted by the manufacturer to the PBAC in Australia and the Committee also considered that obinutuzumab was not cost-effective (PBAC meeting, 2016).

We found that the journal publications considered obinutumab plus bendamustine to be cost-effective compared with bendamustine in R/R-rituximab FL patients, while the HTAs did not.

## 4 Discussion

The rapid review showed that obinutuzumab based chemotherapy significantly prolonged PFS in both patients with untreated FL and those with R/R-rituximab FL. Additionally, the obinutuzumab based chemotherapy regimen also improved OS in patients with R/R-rituximab FL. Two recently published SR/meta-analyses also found that obinutuzumab based chemotherapy regimens significantly prolonged PFS in patients with FL (Leng, 2023; Chu et al., 2024). Furthermore, in terms of PFS prolongation, obinutuzumab plus bendamustine was superior to rituximab plus bendamustine, while there were no significant differences between the obinutuzumab plus CHOP and rituximab plus CHOP regimens, or between obinutuzumab plus CVP and rituximab plus CVP (Wang et al., 2022). The primary data of these studies mainly originated from two open-label, multicenter, randomized phase III clinical trials, GALLIUM and GADOLIN, which introduce less bias into the studies. However, this also leads to a high homogeneity of the results. With the increasing availability of obinutuzumab on the market, there has been a surge in real-world studies, which have also demonstrated favorable effects of obinutuzumab based chemotherapy in prolonging PFS and OS (Zinzani et al., 2023; Bachy et al., 2022; Galusic et al., 2022; Younes et al., 2022; Palomba et al., 2022; Morschhauser et al., 2021; Grigg et al., 2017). In terms of which obinutuzumab based chemotherapy is more advantageous, a study assessing the efficacy and safety of obinutuzumab based chemotherapy as front-line treatment for FL during the COVID-19 pandemic, the results showed that patients treated with obinutuzumab plus CHOP had statistically superior OS and PFS compared with those treated with obinutuzumab plus bendamustine (*p =* 0.002, *p =* 0.006, respectively) (Galusic et al., 2022). Another study found that the PFS of obinutuzumab plus CHOP was shorter than that of obinutuzumab plus bendamustine (Grigg et al., 2017).

Although the included studies showed no advantage of binutuzumab based chemotherapy in improving the ORR of patients, the GAUSS trial (Sehn et al., 2015) suggested that obinutuzumab may induce high ORR than rituximab in FL patients. The GAUSS trial was a randomized, Phase II clinical trial and the first to investigate a head-to-head comparison of obinutuzumab and rituximab in patients with FL. Patients were randomly assigned to receive four doses of either obinutuzumab or rituximab. The ORR assessed by investigators in the FL cohort demonstrated a higher incidence in the obinutuzumab arm (44.6%) than in the rituximab arm (33.3%) (*p* = 0.08, which fell below the pre-specified significance level of 0.2) (Sehn et al., 2015).

In patients with previously untreated FL, the incidence of grade 3–4 AEs, grade 3–4 IRRs, grade 3–4 thrombocytopenia, and grade 3–4 infections was higher in the obinutuzumab based chemotherapy than in the rituximab based chemotherapy. The incidence of all-grade AEs, grade 3–5 AEs, IRRs, neutropenia, and grade 3–5 infections was also higher in patients with R/R-rituximab FL receiving obinutuzumab plus bendamustine than in those receiving bendamustine monotherapy. However, the incidence of thrombocytopenia was higher in the bendamustine monotherapy group than in the obinutuzumab plus bendamustine group. These findings were not consistently observed in other real-world studies. In a retrospective study, the incidence of all-grade thrombocytopenia was higher in the obinutuzumab plus bendamustine group than in the bendamustine monotherapy group, with rates reaching up to 88.9%, including 9 (16.7%) patients with grade 3–4 thrombocytopenia (Fujiwara et al., 2022). Another multicenter retrospective cohort study found that patients in the obinutuzumab and rituximab groups had similar rates of any infections (44.8% and 43.5%, *p* = 1.0) and severe infections (43.3% vs. 47.8%, *p* = 0.844), as well as similar types of infections (Berger et al., 2023). Therefore, in clinical practice, while obinutuzumab can significantly improve PFS and OS, it may increase the incidence of AEs, necessitating close monitoring during treatment.

In terms of economics, there were two pharmacoeconomic studies in China for patients with FL, demonstrating that obinutuzumab based chemotherapy is cost-effective compared with other therapies. The findings were consistent with the journal publications conducted in the US, Italy, Japan and Norway. These studies all demonstrated that when comparing obinutuzumab based chemotherapy to other chemotherapies, there was an increase in overall treatment costs but simultaneous improvement in patients’ QALYs. The ICER was lower than the local Willingness to Pay threshold, making it cost-effective. These cost-effectiveness analyses would benefit from the incorporation of long-term results from the GALLIUM and GADOLIN trials. In the economic evaluation submitted by the manufacturer, national pharmacoeconomic/or drug agencies from different countries all considered that obinutuzumab based chemothrepay should not be cost-effective compared with other chemothrepy regimen (Pan-Candian Oncology Drug Review, 2018; Scottish Medicines Consortium, 2018; Pan-Candian Oncology Drug Review, 2017; PBAC meeting, 2016; National Centre for Pharmacoeconomics, 2018). These economic researches submitted by the manufacturer had limitations, including deviations in outcome measurement and valuation, as well as in the selection of interventions and comparators. The model structure used by the manufacturer, including the choice of exponential distribution for PFS survival probabilities, may have been unreasonable. Furthermore, the assumption of a finite duration of treatment effect on PFS significantly influenced the cost-effectiveness results. Due to these limitations, the evaluation committee (pCODR EGP, SMC, NPCE and PBAC) was driving for a conservative assessment.

## 5 Limitations

This study has several limitations. Firstly, it is a rapid review that conducted a comprehensive evidence narrative analysis of the included studies, which may introduce potential limitations. Secondly, the primary data for the included studies were mainly derived from two open-label, multicenter, randomized phase III clinical trials (GALLIUM and GADOLIN), which could potentially result in a high level of homogeneity in the results. Additional RCTs and real-world studies are needed to evaluate the effectiveness and safety of obinutuzumab. Lastly, only two pharmacoeconomic studies were conducted in China, while the rest were from foreign countries. Further pharmacoeconomic research may be necessary in China to provide evidence.

## 6 Conclusion

Compared with other chemothrapy regimen, obinutuzumab based chemotherapy significantly prolonged PFS and was cost-effective, while its safety profile was considered lower. Therefore, medical professionals should be caution when using or introducing obinutuzumab treatment for FL patients.
