# Supplementary material for: Efficacy, safety and cost-effectiveness of obinutuzumab in patients with follicular lymphoma: a rapid review
Source: Front Pharmacol. 2025 Jan 3;15:1426772. doi: 10.3389/fphar.2024.1426772 (PMC11738910; doi:10.3389/fphar.2024.1426772)
Supplement: Supplementary file 1 [file DataSheet1.docx]

**Appendix**

**Table 1 Quality Evaluation for HTA reports**

| **Item** | **Study ID** | | | | |
| --- | --- | --- | --- | --- | --- |
|  | **pCODR**  **2018** | **SMC**  **2018** | **pCODR**  **2017** | **Ludwig Boltzmann Institute**  **2016** | **PBAC**  **2016** |
| **1. Appropriate contact details for further information?** | YES | YES | YES | YES | NO |
| **2. Authors identified?** | YES | YES | YES | YES | NO |
| **3. Statement regarding conflict of interest?** | YES | NO | YES | YES | NO |
| **4. Statement on whether report externally reviewed?** | YES | NO | YES | NO | NO |
| **5. Short summary in non-technical language?** | YES | YES | YES | YES | YES |
| **6. Reference to the policy question that is addressed?** | YES | YES | YES | YES | NO |
| **7. Reference to the research question(s) that is/are addressed?** | YES | NO | YES | YES | YES |
| **8. Scope of the assessment specified?** | YES | YES | YES | YES | YES |
| **9. Description of the assessed health technology?** | NO | NO | NO | YES | NO |
| **10. Details on sources of information and literature search strategies provided?** | | | | | |
| **10.1 Search strategy** | YES | NO | YES | YES | NO |
| **10.2 Databases** | YES | NO | YES | YES | NO |
| **10.3 Year range** | YES | NO | YES | YES | NO |
| **10.4 Language restriction** | YES | NO | YES | NO | NO |
| **10.5 Primary data** | YES | NO | YES | YES | NO |
| **10.6 Other kind of information resources** | YES | NO | YES | YES | NO |
| **10.7 Complete reference list of included studies** | YES | NO | YES | NO | NO |
| **10.8 List of excluded studies** | NO | NO | NO | NO | NO |
| **10.9 Inclusion criteria** | YES | NO | YES | YES | NO |
| **10.10 Exclusion criteria** | YES | NO | YES | NO | NO |
| **11. Information on basis for the assessment and interpretation of selected data and information?** | | | | | |
| **11.1 Method of data extraction described?** | YES | NO | YES | PARTIAL YES | NO |
| **11.2 Critical appraisal method (for quality assessment of the literature) described?** | YES | NO | YES | NO | NO |
| **11.3 Method of data synthesis described?** | YES | NO | YES | NO | NO |
| **11.4 Results of the assessment clearly presented, e.g. in the form of evidence tables?** | YES | NO | YES | YES | YES |
| **12 (Medico-) legal implications considered?** | YES | YES | YES | YES | NO |
| 1. **Economic analysis provided?** | YES | YES | YES | YES | YES |
| 1. **Ethical implications considered?** | YES | NO | YES | NO | NO |
| 1. **Social implications considered?** | YES | YES | YES | YES | YES |
| 1. **Other perspectives (stakeholders, patients, consumers) considered?** | YES | NO | YES | NO | NO |
| **17. Findings of the assessment discussed?** | YES | NO | YES | YES | YES |
| **18. Conclusions from assessment clearly stated?** | YES | YES | YES | YES | YES |
| **19. Suggestions for further action?** | YES | NO | PARTIAL YES | YES | NO |

**Table 2 Quality Evaluation For SR/Meta-analysis**

| **Items** | **Study ID** | | | | |
| --- | --- | --- | --- | --- | --- |
|  | **Leng WT 2023** | **Chu YR 2023** | **Wang YC 2022** | **Amitai I 2021** | **Police RL 2016** |
| 1.**Did the research questions and inclusion criteria for the review include the components of PICO?** | YES | YES | YES | YES | YES |
| 2.**Did the report of the review contain an explicit statement that the review methods were established prior to the conduct of the review and did the report justify any significant deviations from the protocol?** | PARTIAL YES | PARTIAL YES | YES | PARTIAL YES | PARTIAL YES |
| 3.**Did the review authors explain their selection of the study designs for inclusion in the review?** | YES | YES | YES | YES | YES |
| 4.**Did the review authors use a comprehensive literature search strategy?** | YES | YES | YES | YES | YES |
| **5.Did the review authors perform study selection in duplicate?** | YES | YES | YES | YES | YES |
| **6.Did the review authors perform data extraction in duplicate?** | YES | YES | YES | YES | YES |
| **7.Did the review authors provide a list of excluded studies and justify the exclusions?** | NO | NO | NO | NO | NO |
| **8.Did the review authors describe the included studies in adequate detail?** | YES | YES | YES | YES | YES |
| **9.1 Did the review authors use a satisfactory technique for assessing the risk of bias (RoB) in individual studies that were included in the review?(RCT)** | YES | YES | PARTIAL YES | YES | YES |
| **9.2 Did the review authors use a satisfactory technique for assessing the risk of bias (RoB) in individual studies that were included in the review? (Non-RCT)** | YES |  |  |  |  |
| **10.Did the review authors report on the sources of funding for the studies included in the review?** | NO | YES | YES | YES | YES |
| **11.1 If meta-analysis was performed did the review authors use appropriate methods for statistical combination of results? (RCT)** | YES | YES | YES | YES | YES |
| **11.2 If meta-analysis was performed did the review authors use appropriate methods for statistical combination of results? (non-RCT)** | YES |  |  |  |  |
| 1. **If meta-analysis was performed, did the review authors assess the potential impact of RoB in individual studies on the results of the meta-analysis or other evidence synthesis?** | YES | YES | YES | NO | YES |
| 1. **Did the review authors account for RoB in individual studies when interpreting/ discussing the results of the review?** | YES | YES | YES | NO | YES |
| 1. **Did the review authors provide a satisfactory explanation for, and discussion of, any heterogeneity observed in the results of the review?** | YES | YES | YES | NO | YES |
| 1. **If they performed quantitative synthesis did the review authors carry out an adequate investigation of publication bias (small study bias) and discuss its likely impact on the results of the review?** | YES | NO | NO | YES | NO |
| 1. **Did the review authors report any potential sources of conflict of interest, including any funding they received for conducting the review?** | NO | YES | YES | YES | YES |

**Table3 Quality evaluation of pharmacoeconomic studies**

| **Items** | **Study ID** | | | | | | |  | | |
| --- | --- | --- | --- | --- | --- | --- | --- | --- | --- | --- |
|  | **Ma J**  **2023** | **Wei SD 2022** | **Bellone M 2021** | **Spencer**  **SJ 2021** | **Ohno S 2020** | **Guzauskas**  **GF 2019** | **Haukaas SF**  **2018** | | **Guzauskas GF 2018** | **NCPE2018** |
| 1. **Title** | YES | YES | YES | YES | YES | YES | YES | | YES | YES |
| 1. **Abstract** | YES | YES | YES | YES | YES | YES | YES | | YES | NO |
| 1. **Background and objectives** | YES | YES | YES | YES | YES | YES | YES | | YES | YES |
| 1. **Target population and subgroups** | YES | YES | YES | YES | YES | YES | YES | | YES | YES |
| 1. **Setting and location** | YES | YES | YES | YES | YES | YES | YES | | YES | YES |
| 1. **Study perspective** | YES | YES | YES | YES | YES | YES | YES | | YES | YES |
| 1. **Comparators** | YES | YES | YES | YES | YES | YES | YES | | YES | YES |
| 1. **Time horizon** | YES | YES | YES | YES | YES | YES | YES | | YES | YES |
| 1. **Discount rate** | YES | YES | YES | YES | YES | YES | YES | | YES | NO |
| 1. **Choice of health outcomes** | YES | YES | YES | YES | YES | YES | YES | | YES | YES |
| 1. **Measurement of effectiveness** | YES | YES | YES | YES | YES | YES | YES | | YES | YES |
| 1. **Measurement and valuation of preference-based outcomes** | YES | PARTIAL YES | YES | YES | YES | YES | YES | | YES | YES |
| 1. **Estimating resources and costs** | YES | YES | YES | YES | YES | YES | YES | | YES | YES |
| 1. **Currency, price date, and conversion** | YES | YES | YES | YES | YES | YES | YES | | YES | NO |
| 1. **Choice of model** | YES | YES | YES | YES | YES | YES | YES | | YES | YES |
| 1. **Assumptions** | TES | YES | YES | YES | YES | YES | YES | | YES | YES |
| 1. **Analytical methods** | YES | YES | YES | YES | YES | YES | YES | | YES | NO |
| 1. **Study parameters** | YES | YES | YES | YES | YES | YES | YES | | YES | NO |
| 1. **Incremental costs and outcomes** | YES | YES | YES | YES | YES | YES | YES | | YES | YES |
| 1. **Characterising uncertainty** | YES | YES | YES | YES | YES | YES | YES | | YES | NO |
| 1. **Characterising**   **heterogeneity** | YES | YES | YES | YES | YES | YES | YES | | YES | NO |
| 1. **Study findings,**   **limitations,**  **generalisability, and current knowledge** | YES | YES | YES | YES | YES | YES | YES | | YES | NO |
| 1. **Source of funding** | YES | NO | YES | YES | YES | YES | YES | | YES | NO |
| 1. **Conflicts of interest** | YES | NO | YES | YES | YES | YES | YES | | YES | NO |
